# A novel cysteine protease inhibitor in *Baylisascaris schroederi* migratory larvae regulates inflammasome activation through the TLR4–ROS–NLRP3 pathway

**DOI:** 10.1186/s13071-022-05466-6

**Published:** 2022-09-23

**Authors:** Jingyun Xu, Xiaobin Gu, Yue Xie, Ran He, Jing Xu, Lang Xiong, Xuerong Peng, Guangyou Yang

**Affiliations:** 1 Department of Parasitology, College of Veterinary Medicine, Sichuan Agricultural University, Wenjiang, 611130 China; 2 Department of Chemistry, College of Life and Basic Science, Sichuan Agricultural University, Wenjiang, 611130 China

**Keywords:** *Baylisascaris schroederi*, Cysteine protease inhibitors, Inflammasome, ROS, Pyroptosis

## Abstract

**Background:**

Giant pandas (*Ailuropoda melanoleuca*) are the obligate host of the parasitic roundworm *Baylisascaris schroederi*. The infection of giant pandas with *B. schroederi* is very common. At present, little is known about the mechanism of immune interaction between *B. schroederi* and the host. As an important component of innate immunity, the NOD-like receptor 3 (NLRP3) inflammasome plays an important role in host immune response and the occurrence and development of infectious diseases.

**Methods:**

We analyzed the regulation of NLRP3 inflammasome activation in monocyte-derived macrophages (MDMs) by the recombinant* B. schroederi* migratory larvae cysteine protease inhibitor rBsCPI-1, knowing from a previous study that the CPI-1 is highly expressed in *B. schroederi* migratory larvae. We first determined the effects of rBsCPI-1 and excretory–secretory products of *B. schroederi* migratory larvae on cell proliferation using the CCK-8 and LDH release assays. We then analyzed NLRP3 inflammasome activation, pyroptosis and pro-inflammatory cytokine release by quantitative-PCR, western blotting and enzyme-linked immunosorbent assay. The signaling pathway of rBsCPI-1 to activate NLRP3 inflammasomes was analyzed in activation and inhibition experiments. Finally, the effects of rBsCPI-1 on inflammasome activation in mice immunized with rBsCPI-1 were analyzed.

**Results:**

The activation and inhibition experiments revealed that rBsCPI-1 induced inflammasome activation through the TLR4–ROS–NLRP3 signaling pathway, with reactive oxygen species (ROS) not only functioning as an activator of the NLRP3 inflammasome, but also an activation product of the NLRP3 inflammasome. rBsCPI-1 promoted the activation and assembly of the NLRP3 inflammasome, which further converted the pro-inflammatory cytokines interleukin (IL)-1β and IL-18 into mature active forms. At the same time, caspase-1 cleaved gasdermin D to trigger cell pyroptosis. The results of animal immunization experiments further confirmed that rBsCPI-1 could induce the activation of the NLRP3 inflammasome.

**Conclusions:**

rBsCPI-1 activates the inflammasome through the TLR4–ROS–NLRP3 signaling pathway and further induces the pyroptosis of MDMs and release of pro-inflammatory factors IL-1β and IL-18, thus promoting the occurrence and development of the inflammatory response in the host.

**Graphical abstract:**

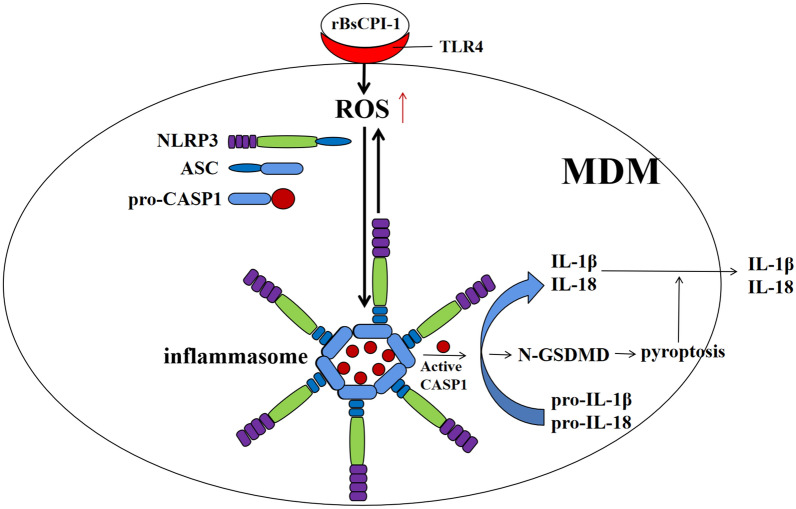

## Background

Baylisascariasis is a common and serious parasitic disease of giant pandas (*Ailuropoda melanoleuca*) caused by infection with adults or migratory larvae of the parasitic roundworm *Baylisascaris schroederi*. The adult roundworm usually resides in the intestines of giant pandas and can cause intestinal inflammation, intestinal blockage and even lethal intestinal rupture. The migratory larvae can migrate to various organs of giant pandas and induce visceral larva migrans (VLM), causing severe intestinal damage, helminthic hepatitis, pneumonia and acute pancreatitis, all of which have a great impact on the survival and health of giant pandas [1–4].

The structure and antigen components of parasites are more complex than those of bacteria and viruses and, consequently, it is extremely difficult to elucidate the complete immune interaction mechanism between parasites and hosts. Current research focuses on analyzing whether a specific protein in the excretory–secretory products plays an immunoregulatory role. Experimental data have shown that cysteine protease inhibitors (CPIs) play an important role in the regulation of host immune responses [5, 6]. The CPI secreted by worms may have a dual function of both regulating intrinsic proteases and inhibiting immune-related host’s proteases [7–10]. In a previous study, we cloned, expressed and purified recombinant* B. schroederi* migratory larvae CPI (rBsCPI-1), which was highly expressed in *B. schroederi* migratory larvae, and analyzed its regulation of the function of peripheral blood mononuclear-derived macrophages (MDMs). We found that rBsCPI-1 can exert a dual regulatory role on mouse immune cells through the recognition of Toll-like receptor 4 (TLR4) (unpublished results).

Inflammation is an important response of the host immune system to modulate defense mechanisms and control microbial invasion. However, prolonged or unrelieved inflammation increases the risk of inflammatory disease development and, therefore, precise control of the inflammatory response is essential for limiting pathogen infection without causing damage to the host. The inflammasome is a multi-protein complex which is an important component of the innate immune system. Activation of the inflammasome is initiated by pattern recognition receptors sensing pathogen-associated molecular patterns (PAMPs) or damage-associated molecular patterns (DAMPs) and upregulating NOD-like receptor 3 (NLRP3) expression [11]. Intracellular NLRP3 can recruit apoptosis-associated speck-like protein-containing CARD (ASC) and caspase-1 precursor to form the NLRP3 inflammasome, thereby inducing the maturation and secretion of interleukin (IL)-1β and IL-18 [12]. Previous studies have shown that infection with *Schistosoma mansoni*, *Toxoplasma gondii* and *Trypanosoma cruzi* can activate the NLRP3 inflammasome in host cells [13–15]. *Plasmodium vivax* and *Toxoplasma gondii* have also been shown to activate various inflammasomes [16, 17].

Although research on the inflammasome has become a hot spot, there have been no relevant studies on the relationship between *B. schroederi* infection and inflammasome activation. It also remains to be explored whether rBsCPI-1 regulates the occurrence and development of inflammatory responses by activating the NLRP3 inflammasome. The findings of the study reported here will provide insights into the pathogenesis of baylisascariasis and also help in the conservation of the giant panda species.

## Methods

### Experimental animals and cells

Specific pathogen-free female BALB/c mice (*n* = 40 in total), aged approximately 6–8 weeks, were purchased from Chengdu Dashuo Laboratory Animal Co., Ltd. (Chengdu, China) The animal study protocol was reviewed and approved by the Animal Care and Use Committee of Sichuan Agricultural University (no.: SYXK 2019-189). All animal procedures used in this study were performed in accordance with the Guide for the Care and Use of Laboratory Animals (National Research Council, Bethesda, MD, USA) and recommendations of the Animal Research: Reporting of In Vivo Experiments (ARRIVE) guidelines (http://www.nc3rs.org.uk/arrive-guidelines). All applicable institutional and national guidelines for the care and use of animals were followed.

Peripheral blood mononuclear cells (PBMCs) were separated using the Mouse Peripheral Blood Mononuclear Cell Separation Solution Kit (TBD, China) as previously described [18]. The PBMCs (1 × 10^6^/ml) were cultured at 37 °C and 5% CO_2_ in RPMI 1640 complete medium (HyClone Laboratories LLC, Logan, UT, USA) supplemented with 2 mM l-glutamine, 50 ng/ml M-CSF (Solarbio Science & Technology Co., Ltd., Beijing, China), and 10% fetal bovine serum (Sigma-Aldrich, St. Louis, MO, USA). The medium was changed every 2 days until monocytes differentiated and proliferated sufficiently. Approximately 1 week of culture was needed to obtain MDMs [19].

### Protein preparation

The excretory–secretory products of *B. schroederi* migratory larvae (BsES) were prepared as previously described [18]. The pET-32a-BsCPI-1 positive expression bacterial solution was expressed and purified as reported previously [18]. The protein concentrations of the prepared BsES and rBsCPI-1 were determined by the bicinchoninic acid (BCA) assay (Takarabio, Beijing, China). The contaminated endotoxin was removed by the ToxOut™ High Capacity Endotoxin Removal Kit (GenScript Biotech, China).

### Cell proliferation assay

Approximately 100 µl MDM suspension (1 × 10^6^/ml) was treated with concanavalin A (ConA: 10 µg/ml) alone or in combination with pET-32a, different concentrations of rBsCPI-1 (0, 5, 10, 15, 30, 50 µg/ml) or different concentrations of BsES (0, 1, 5, 10, 20, 30 µg/ml), in the same volume for 12 h. Next, 10 µl Cell Counting Kit 8 (CCK-8; Meilunbio, Dalian, China) solution was added to each well and incubated for 4 h before harvesting; the absorbance values were measured at 450 nm.

### Lactate dehydrogenase cytotoxicity assay

Approximately 100 µl MDM suspension (1 × 10^6^/m) was treated with phosphate-buffered saline (PBS), pET-32a, rBsCPI-1 or BsES for 12 h, following which the supernatant was collected and 150 µl of lactate dehydrogenase (LDH) release reagent was added to the supernatant at a 10× dilution in PBS. Following mixing, the solution was incubated at 37 °C and 5% CO_2_ for 1 h, then 120 µl of supernatant was collected and the absorbance values measured at 490 nm

### Quantitative PCR analysis of inflammasome activation

Approximately 100 µl MDM suspension (1 × 10^6^/ml) was treated respectively with PBS, pET-32a, rBsCPI-1, BsES and Nigericin sodium salt, a NLRP3 activator (10 μM; MedChemExpress, Monmouth Junction, NJ, USA). Quantitative PCR (qPCR) was used to analyze the transcription level of the NLRP3, AIM2, ASC, caspase-1, caspase-11, IL-1β and IL-18 genes using the primers shown in Table 1. Total RNA was extracted from MDMs using the Total RNA Extraction Kit (Solarbio). Complementary DNA (cDNA) was synthesized from total RNA using the Prime Script First Strand cDNA Synthesis Kit (Thermo Fisher Scientific, Waltham, MA, USA), and qPCR reactions were performed using Roche LightCycler 96 real-time PCR cycler (Roche, Basel, Switzerland). Amplifications were conducted in a 20-μl reaction volume containing 10 μl of TB Green Premix Ex Taq™ (Tli RNase H Plus; TaKaRa, Kusatsu, Japan), 2 μl of cDNA template (50 ng), 0.8 μl each of forward and reverse primer (10 μM) and 6.4 μl of ddH_2_O. The PCR amplification program consisted of 95 °C for 10 min; 40 cycles of 95 °C for 5 s, 60 °C for 30 s; and 95 °C for 5 s, 60 °C for 60 s, 95 °C for 1 s. Transcription levels of the target genes were normalized by subtracting the expression level of glyceraldehyde 3-phosphate dehydrogenase (GAPDH) and then calculating the relative expression using the 2^−∆∆Ct^ method.

**Table 1:** Primers of the detected genes

Genes	Primers	Sequence	Accession no.
Nlrp1α	Forward	5ʹ-ACATCCACATACTGCTCAC-3ʹ	NM_001004142
	Reverse	5ʹ-ATCTTCACACCACCATCAC-3ʹ	
Nlrp3	Forward	5ʹ-AGACCTCCAAGACCACTAC-3ʹ	BC116175
	Reverse	5ʹ-ACATAGCAGCGAAGAACTC-3ʹ	
Nlrp6	Forward	5ʹ-GGTGAAGGAGAGGAATGC-3ʹ	NM_133946
	Reverse	5ʹ-GGATGAACAGTAGGCGATT-3ʹ	
Nlrc4	Forward	5ʹ-GCTCAGTCCTCAGAACCT-3ʹ	NM_001033367
	Reverse	5ʹ-GCCACCAACTTCATCTCTT-3ʹ	
Aim2	Forward	5ʹ-CAGTTCCTCAGTTGTGGTT-3ʹ	NM_001013779
	Reverse	5ʹ-GTCTAATCTTGTCTCCTTCCT-3ʹ	
Asc	Forward	5ʹ-CACAGGCAAGCACTCATT-3ʹ	NM_023258
	Reverse	5’-CAGGTCAGGTTCCAGGAT-3’	
Caspase-1	Forward	5ʹ-GACATCCTTCATCCTCAGAA-3ʹ	NM_009807
	Reverse	5ʹ-CTCCAGCAGCAACTTCAT-3ʹ	
Caspase-11	Forward	5ʹ-CCATAGTGCTGCTCATCC-3ʹ	NM_175362
	Reverse	5ʹ-CGCTGCTCCTCTACAATC-3ʹ	
IL-1b	Forward	5ʹ-CTTCAGGCAGGCAGTATC-3ʹ	NM_008361
	Reverse	5ʹ-CAGCAGGTTATCATCATCATC-3ʹ	
IL-18	Forward	5ʹ-ACTCTTGCGTCAACTTCA-3ʹ	BC024384
	Reverse	5ʹ-GTCCTCTTACTTCACTGTCT-3ʹ	
GAPDH	Forward	5ʹ-TCTCCTGCGACTTCAACA-3ʹ	GU214026
	Reverse	5ʹ-TGTAGCCGTATTCATTGTCA-3ʹ	

### Western blotting analysis of inflammasome activation

Approximately 100 µl MDM suspension (1 × 10^6^/ml) was treated with PBS, pET-32a, rBsCPI-1, BsES and Nigericin sodium salt, respectively. The cells were collected, and the MDM proteins were prepared as mentioned previously [18]. A total of 100 µg MDM proteins was separated using sodium dodecyl sulfate-polyacrylamide gel electrophoresis, and the separated proteins were blotted onto nitrocellulose filter membranes. The membranes were then blocked with 5% skim milk for 2 h at room temperature, following which they were incubated with Anti-ASC, Anti-cleaved N-terminal GSDMD, Anti-IL-1 beta and Anti-IL-18 (1:1000; diluted in 5% skim milk; ABclonal, Wuhan, China) at 4 °C overnight. After three washes, the membranes were incubated with horseradish peroxidase (HRP)-conjugated secondary antibody (1:5000; diluted in 5% skim milk; ABclonal) for 2 h at room temperature. After washing, the membranes were exposed using ultrasensitive ECL chemiluminescence reagent (Meilunbio). The bands were quantified using densitometry and analyzed with Image J software.

Approximately 100 µl MDM suspension (1 × 10^6^/ml) was pre-incubated with 1 mM *N*-acetylcysteine (NAC; a reactive oxygen species [ROS] inhibitor; MedChemExpress) for 4 h, following which rBsCPI-1 was added and the solution incubated for 12 h. The cells were then collected, and the MDM proteins were prepared and western blotting carried out as described above. Anti-NLRP3 (ABclonal) was diluted 1:1000 in 5% skim milk.

Approximately 100 µl MDM suspension (1 × 10^6^/ml) was pre-incubated with 10 µM MCC950 Sodium (a NLRP3 inhibitor; MedChemExpress) for 4 h, following which rBsCPI-1 was added and the solution incubated for 12 h. The cells were then collected, and MDM proteins were prepared and western blotting was carried out as mentioned above. Anti-caspase1, Anti-GSDMD, Anti-cleaved N-terminal GSDMD, Anti-IL-1 beta and Anti-IL-18 (ABclonal) were each diluted 1:1000 in 5% skim milk.

### Enzyme-linked immunosorbent assay for cytokine expression

Approximately 100 µL MDM suspension (1 × 10^6^/ml) was treated with PBS, pET-32a, rBsCPI-1, BsES and Nigericin sodium salt, respectively. Cell culture supernatant were collected by centrifugation for 15 min at 1000 *g*, following which 100 μl of standard and sample was added to each well, and the wells incubated for 2 h at 37 °C. After incubation, the liquid was removed from each well, 100 μl of biotin antibody (1×) was added and the wells incubated for 1 h at 37 °C, followed by three washes. HRP-avidin (100 μl; 1×) was added to each well and the wells incubated for 1 h at 37 °C. After washing, 100 μl of TMB Substrate was added to each well and the wells incubated for 30 min. Finally, 50 μl of Stop Solution was added to each well and the absorbance values were measured at 450 nm.

### ROS assay

The TLR-4 on MDMs were pre-activated or pre-blocked with lipopolysaccharide (LPS; a TLR4-specific activator; Sigma-Aldrich) or 7E3 (a TLR-4-blocking antibody; Abcam, Cambridge, UK) for 24 h, and then rBsCPI-1 was added and incubated for 12 h. The fluorogenic dye 2′-7′dichlorofluorescin diacetate (DCFH-DA; Solarbio) was diluted 1:1000 with serum-free medium to a final concentration 10 μM/l. MDMs were incubated with DCFH-DA at 37 °C, 5% CO_2_ for 20 min, then washed three times with serum-free cell culture medium to sufficiently removed DCFH-DA that did not enter the cells. The cells were then collected and detected by measurement fluorescence at 488 nm excitation and 525 nm emission wavelengths.

MDMs were pre-incubated with 10 µM Nigericin or MCC950 sodium for 4 h. rBsCPI-1 was then added and the solution incubated for 12 h. ROS analysis experiments were then carried out.

### In vivo experiment

A total of 18 mice were divided into three groups of six mice each, and each group of six mice injected via the intraperitoneal route with 50 µg rBsCPI-1, an equal volume of PBS or pET-32a, for a total of three times at 7-day intervals. The mice were then sacrificed 7 days after the last immunization, and MDMs were collected. The relative expression of the NLRP3, AIM2, ASC, caspase-1, caspase-11, IL-1β and IL-18 genes were measured using the same qPCR instrumentation and protocols as described above.

### Statistical analysis

All data are expressed as mean ± standard deviation (SD). Statistical analysis was done using GraphPad Prism 5 software (GraphPad Software, San Diego, CA, USA. Image J software was used to quantify the protein band intensity. Differences between groups were assessed by one-way analysis of variance in SPSS version 26.0 software (SPSS IBM Corp., Armonk, NY, USA. *P* < 0.05 was considered to indicate statistical significance.

## Results

### rBsCPI-1 affected cell proliferation

**Fig. 1:**
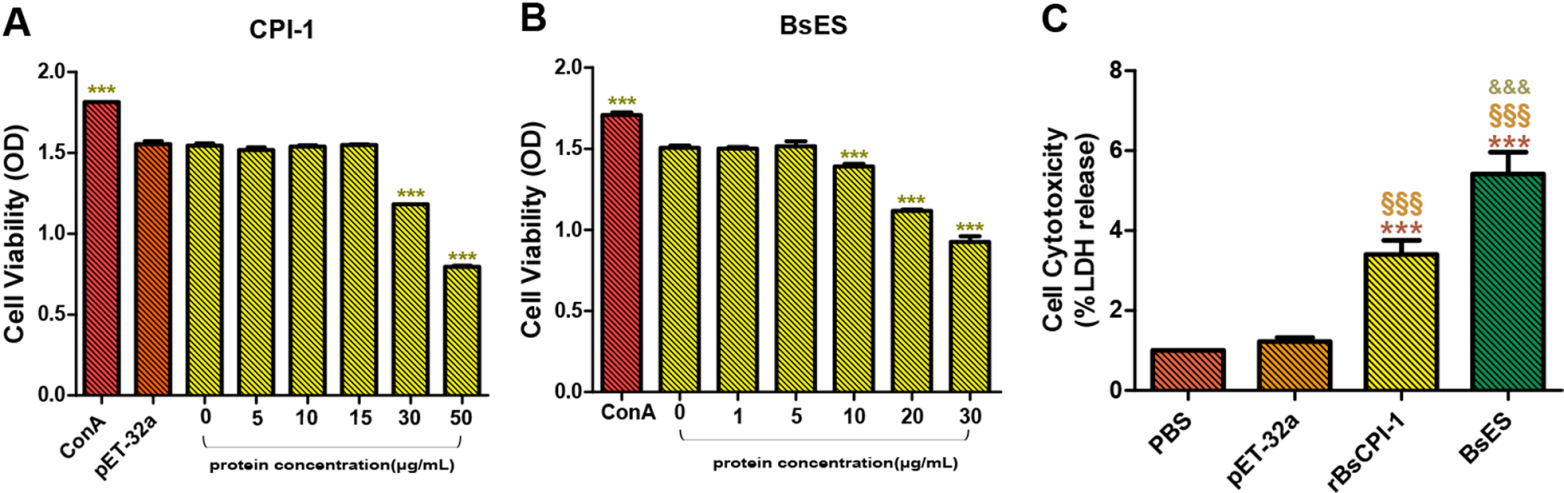
rBsCPI-1 and BsES affect the proliferation of monocyte-derived macrophages (MDMs).**A**, **B** Proliferation was measured by the Cell Counting Kit 8 (CCK-8) assay after the cells were stimulated with ConA alone or combined with rBsCPI-1 (**A**) and BsES (**B**). The optical density (OD) values at 450 nm were considered to be the cell proliferation index. The data from 3 independent experiments were analyzed and expressed as the mean ± standard deviation (SD). Asterisks indicate a significant difference compared with the 0 μg/ml group at *P < 0.05, **P < 0.01, ***P < 0.001.** c** The cell cytotoxicity assay was used to assess the release level of LDH at OD_490_. Significant differences versus the PBS group are indicated by ***P < 0.001; versus the pET-32a (pET-32a) group by ^§§§^*P* < 0.001; and versus the rBsCPI-1 group by ^&&&^*P* < 0.001. BsES, Excretory–secretory products of *Baylisacaris schroederi* migratory larvae; ConA, Concanavalin A; LDH, lactate dehydrogenase; PBS, phosphate-buffered saline; rBsCPI-1, recombinant* B. schroederi* migratory larvae cysteine protease inhibitor

In order to analyze the effect of rBsCPI-1 on cell proliferation and determine the optimal reaction concentration of rBsCPI-1 and BsES co-incubated with MDMs, we performed cell proliferation experiments using the CCK-8 kit. As shown in Fig. 1a, b, the proliferation ability of MDMs gradually decreased with increasing concentrations of rBsCPI-1 and BsES until the rBsCPI-1 concentration reached 30 μg/ml (*P* < 0.001) and the BsES concentration reached 10 μg/ml (*P* < 0.001), at which levels the proliferation of MDMs was significantly inhibited. Therefore, in order to exclude the effect of cell proliferation on subsequent experiments, we selected 15 μg/ml rBsCPI-1 and 5 μg/ml BsES as the optimal reaction concentrations for co-incubation with MDMs.

At the same time, we analyzed the cell cytotoxicity of rBsCPI-1 and BsES on MDMs by detecting the release of LDH. The results showed that the release of LDH was significantly increased in the rBsCPI-1 and BsES group compared with the control and pET-32a group (*P* < 0.001). Also, there was also difference between the rBsCPI-1 and the BsES group (*P* < 0.001) (Fig. 1c). We therefore concluded that rBsCPI-1 and BsES have a cell cytotoxic effect on MDMs.

### rBsCPI-1 and BsES activated the NLRP3 inflammasome

**Fig. 2:**
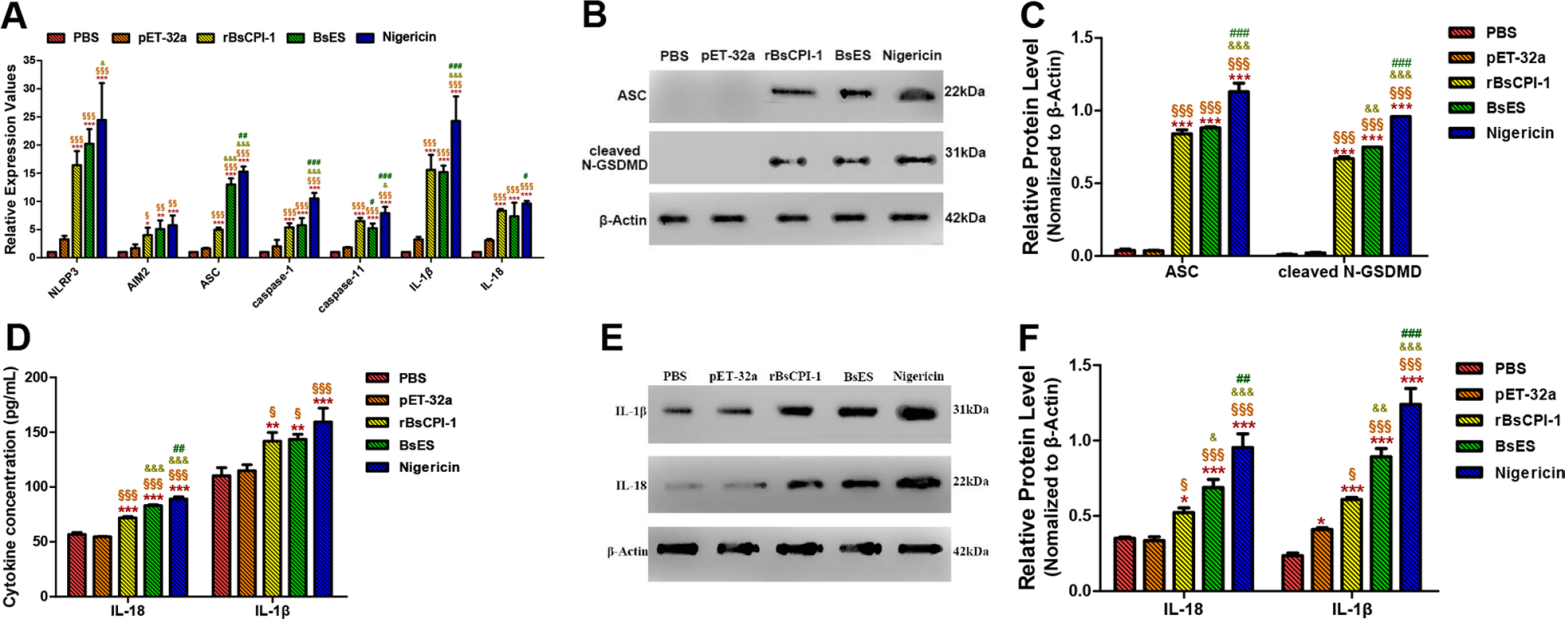
rBsCPI-1 and BsES induced inflammasome activation.** A** Quantitative PCR analysis of the transcription levels of the NOD-like receptor 3 (NLRP3) inflammasome-related genes in MDMs. **B**, **C** A representative western blot band of ASC and cleaved N-terminal gasdermin D (GSDMD) are shown in **B**, and a graph of the quantified band density is shown in **C**. **D** The enzyme-linked immunosorbent assay (ELISA) detected the secreted IL-1β and IL-18 in the cell supernatant. **E**, **F** A representative western blotting band of retained IL-1β and IL-18 in the cells are shown in **E**, and the graph of the quantified band density is shown in **F**. Data are shown as mean ± SD of 3 replicates per group. Significant differences versus the control group are indicated by *P < 0.05, **P < 0.01, ***P < 0.001; versus pET-32a group by ^§^*P* < 0.05, ^§§^*P* < 0.01, ^§§§^*P* < 0.001; versus the rBsCPI-1 group by ^&^*P* < 0.05, ^&&^*P* < 0.01, ^&&&^*P* < 0.001; and versus the BsES group by ^#^*P* < 0.05, ^##^*P* < 0.01, ^###^*P* < 0.001. GSDMD, Gasdermin D; IL, interleukin

The activation of the NLRP3 inflammasome in MDMs under the effects of PBS, pET-32a, rBsCPI-1, BsES and Nigericin was analyzed by qPCR. The experimental results showed that pET-32a did not cause inflammasome activation compared with the control group, while BsES caused significant increases in the transcript levels of the NLRP3, AIM2, ASC, caspase-1, caspase-11, IL-1β and IL-18 genes. Meanwhile, rBsCPI-1 caused a significant increase in the transcript levels of these genes compared to the levels in the control group and a significant decrease compared to those in the Nigericin-positive group. With the exception of ASC, there was no significant difference between BsES and rBsCPI-1 in inducing a significant increase in the transcription levels of the other genes (Fig. 2a).

Western blotting results showed that ASC was significantly activated by rBsCPI-1, BsES and Nigericin compared with the control group (*P* < 0.001). Meanwhile, rBsCPI-1, BsES and Nigericin promoted gasdermin D (GSDMD) cleavage in response to canonical and non-canonical inflammasome activators (Fig. 2b, c).

At the same time, we detected the level of secreted (in the supernatant) and retained (in the cell) cytokines using the enzyme-linked immunosorbent assay (ELISA) (Fig. 2d) and western blotting (Fig. 2e, f). The results showed that the level of IL-1β and IL-18 secreted and retained were all significantly higher in the rBsCPI-1, BsES, and Nigericin groups compared with the control and pET-32a groups. Also, the levels of cytokines in the Nigericin-positive group were significantly higher than those in the rBsCPI-1 and BsES groups. Based on these results, we drew a preliminary conclusion that rBsCPI-1 and BsES can induce the activation of inflammasomes in MDMs.

### TLR4 induced ROS production

**Fig. 3:**
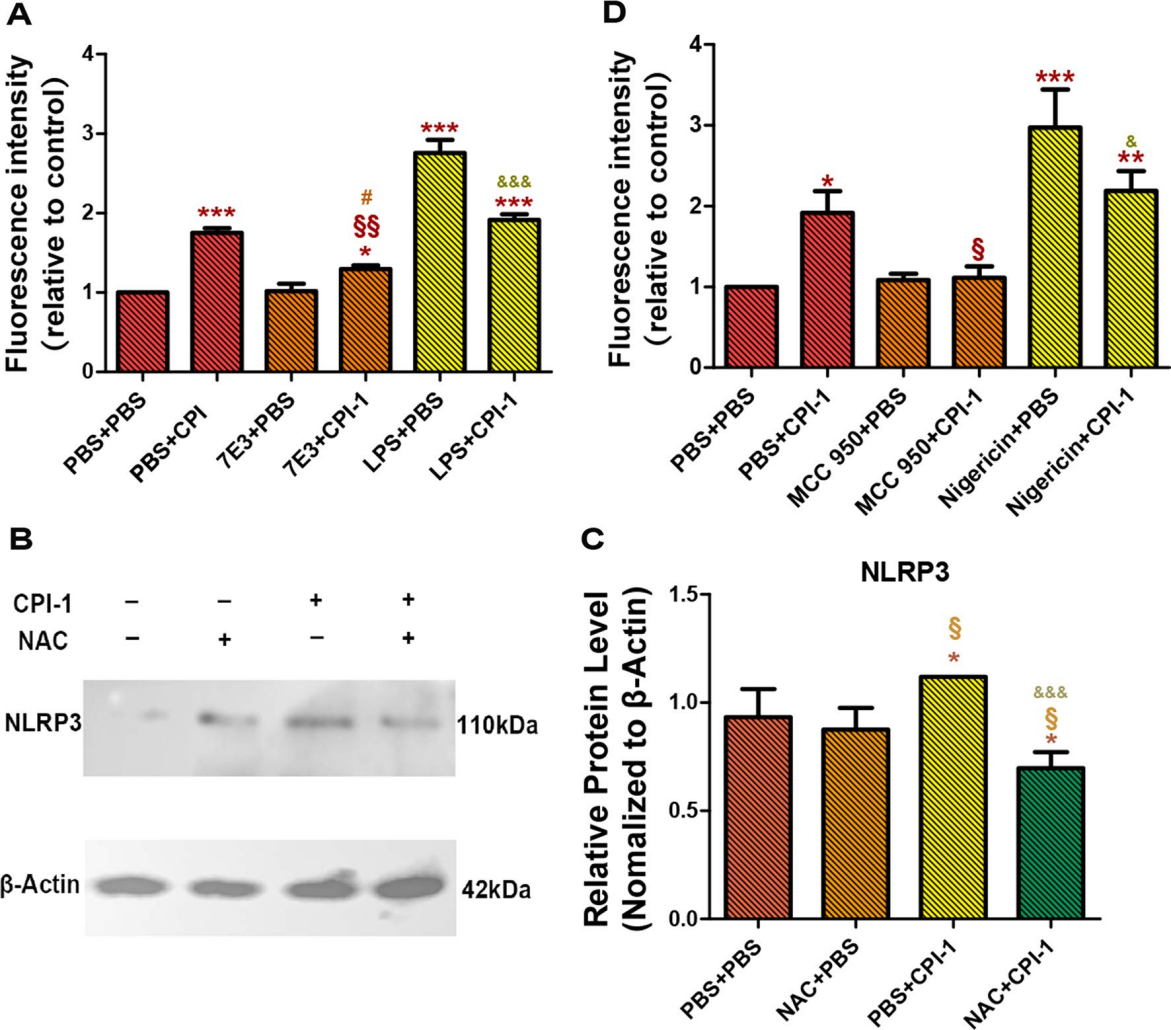
rBsCPI-1 affects NLRP3 activation through the TLR4-ROS-NLRP3 pathway. **A** The fluorescence intensity of intracellular fluorogenic dye 2′-7′dichlorofluorescin diacetate (DCFH-DA) was detected at 488 nm excitation and 525 nm emission wavelengths. The data presented are representative of 3 independent experiments. Significant differences versus the control group (PBS + PBS) are indicated by *P < 0.05, ***P < 0.001; for the LPS + CPI-1 group versus the LPS + PBS group by ^&&&^*P* < 0.001; for the 7E3 + CPI-1 group versus the 7E3 + PBS group by ^#^*P* < 0.05; and for the LPS + CPI-1 group versus the LPS + PBS group by ^&&^*P* < 0.01. **B**, **C** A representative western blot is shown in **B**, and the graph of the quantified band density is shown in **C**. C *P < 0.05; versus the NAC + PBS group by ^§^*P* < 0.05; versus PBS + CPI-1 group by ^&&&^*P* < 0.001. **D** Fluorescence intensity of intracellular DCFH-DA. Significant differences versus the control group are indicated by *P < 0.05, **P < 0.01, ***P < 0.001; for the MCC 950 + CPI-1 group or Nigericin + CPI-1 group versus the PBS + CPI-1 group by ^§^*P* < 0.05; for the Nigericin + CPI-1 group versus the Nigericin + PBS group by ^&^*P* < 0.05. 7E3, TLR-4-blocking antibody; LPS, lipopolysaccharide (TLR4 activator); MCC 950, MCC950 Sodium (a NLRP3 inhibitor); NAC, *N*-acetylcysteine (a ROS inhibitor); ROS, reactive oxygen species; TLR4, Toll-like receptor 4

We pre-incubated MDMs with TLR4 activator (LPS) and TLR4 inhibitor (7E3) for 24 h, then added rBsCPI-1 for stimulation. The fluorescence intensity of intracellular DCFH-DA was detected to analyze the expression of ROS. The experimental results showed that rBsCPI-1 induced a high expression of ROS and that there was no significant difference in ROS expression between the 7E3 + PBS groups and the control group (*P* > 0.05). The expression of ROS in the LPS + PBS and LPS + CPI-1 groups (*P* < 0.001) were significantly higher than that in the control group, and the expression of ROS in the LPS + CPI-1 group was significantly lower than that in the LPS + PBS group (*P* < 0.001) (Fig. 3a).

### ROS induced NLRP3 activation

We pre-incubated MDMs with the ROS inhibitor (NAC) for 4 h and then added rBsCPI-1 for stimulation. NLRP3 activation was assessed by western blotting. The results showed that under the action of rBsCPI-1, the expression of NLRP3 in MDMs was significantly higher than that in the control group (*P* < 0.05). The addition of rBsCPI-1 after pre-incubation with NAC could induce an decrease in the expression of NLRP3 compared with the control (*P* < 0.05) and PBS + CPI-1 group (*P* < 0.001) groups (Fig. 3b, c).

### NLRP3 activation induced ROS production

We pre-incubated MDMs with NLRP3 activator (Nigericin) and NLRP3 inhibitor (MCC950) for 4 h and then added rBsCPI-1 for stimulation. The fluorescence intensity of the intracellular DCFH-DA was detected to analyze the expression of ROS. The results showed that rBsCPI-1 induced a high expression of ROS (*P* < 0.05), but at a lower level than that induced by Nigericin. The expression of ROS in the Nigericin + CPI-1 group was significantly lower than that in the Nigericin + PBS group (*P* < 0.05). On the other hand, there was no significant difference in ROS expression when the MCC 950 + PBS and MCC 950 + CPI-1 groups were compared with the control group, while the expression in the MCC950 + CPI-1 group was significantly lower than that in the PBS + CPI-1 group (*P* < 0.05) (Fig. 3d).

### rBsCPI-1 induced cell pyroptosis

**Fig. 4:**
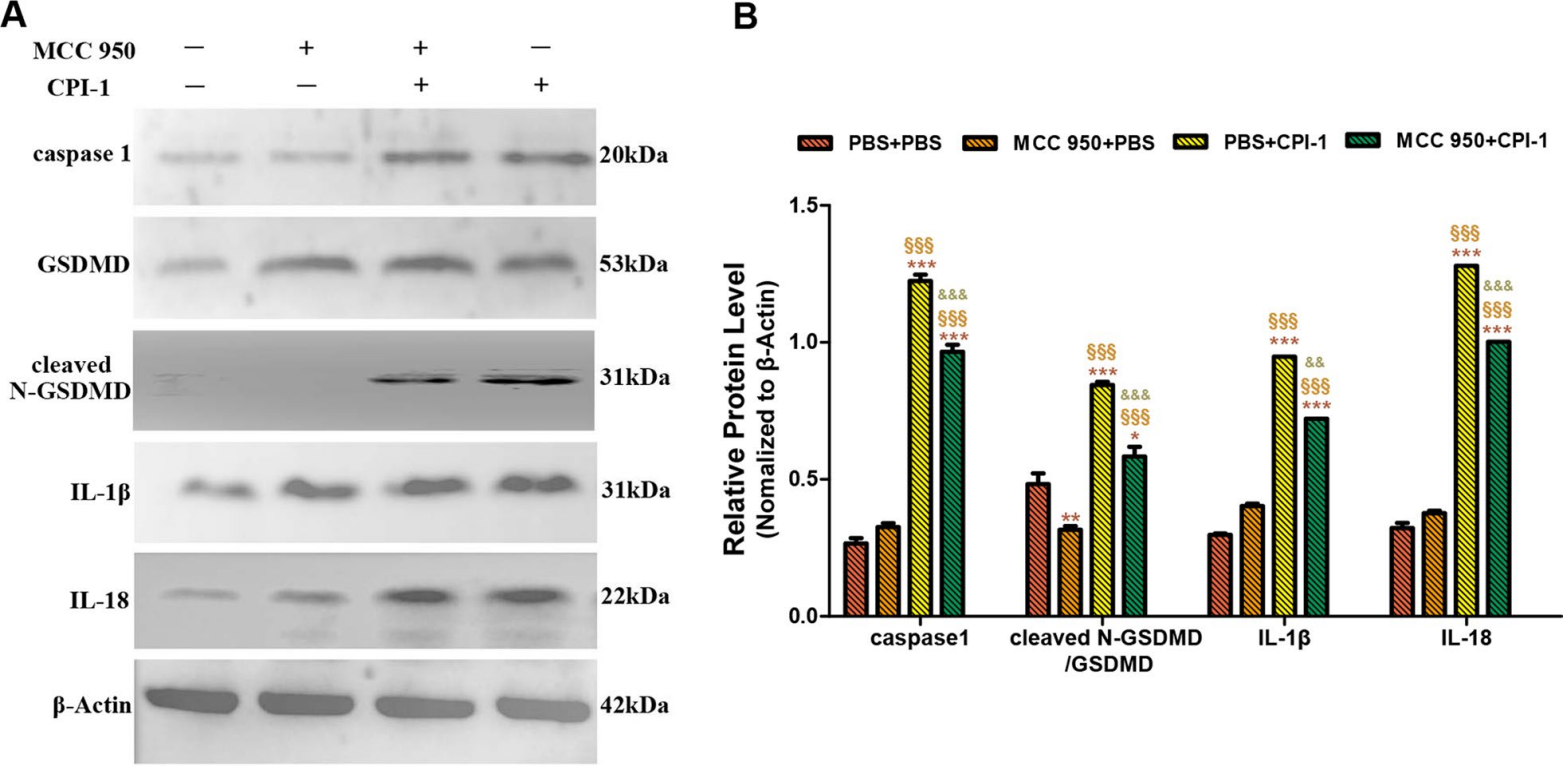
The activation of NLRP3 inflammasome-induced pyroptosis. a b A representative western blotting is shown in **A**, and the graph of the quantified band density is shown in **B**. Data are shown as the mean ± SD of 3 replicates per group. Significant differences versus the control group are indicated by *P < 0.05, **P < 0.01, ***P < 0.001; versus the MCC 950 + PBS group by ^§^*P* < 0.05; versus the PBS + CPI-1 group by ^&^*P* < 0.05, ^&&&^*P* < 0.001

The expression of pyroptosis-related proteins was detected by western blotting. The experimental results showed that under the action of rBsCPI-1, the expression levels of the pyroptosis-related proteins caspase-1, cleaved* N*-terminal GSDMD, IL-1β and IL-18 were significantly higher than those in the control group (*P* < 0.001). At the same time, when MDMs were pre-incubated with the NLRP3 inhibitor MCC950, the expression levels of caspase-1, IL-1β and IL-18 were not significantly different from those in the control group. The expression levels of caspase-1, cleaved* N*-terminal GSDMD, IL-1β and IL-18 in the MCC950 + CPI-1 group were significantly higher than those in the control group, and significantly lower than those in the PBS + CPI-1 group (Fig. 4).

### In vivo experiment

**Fig. 5:**
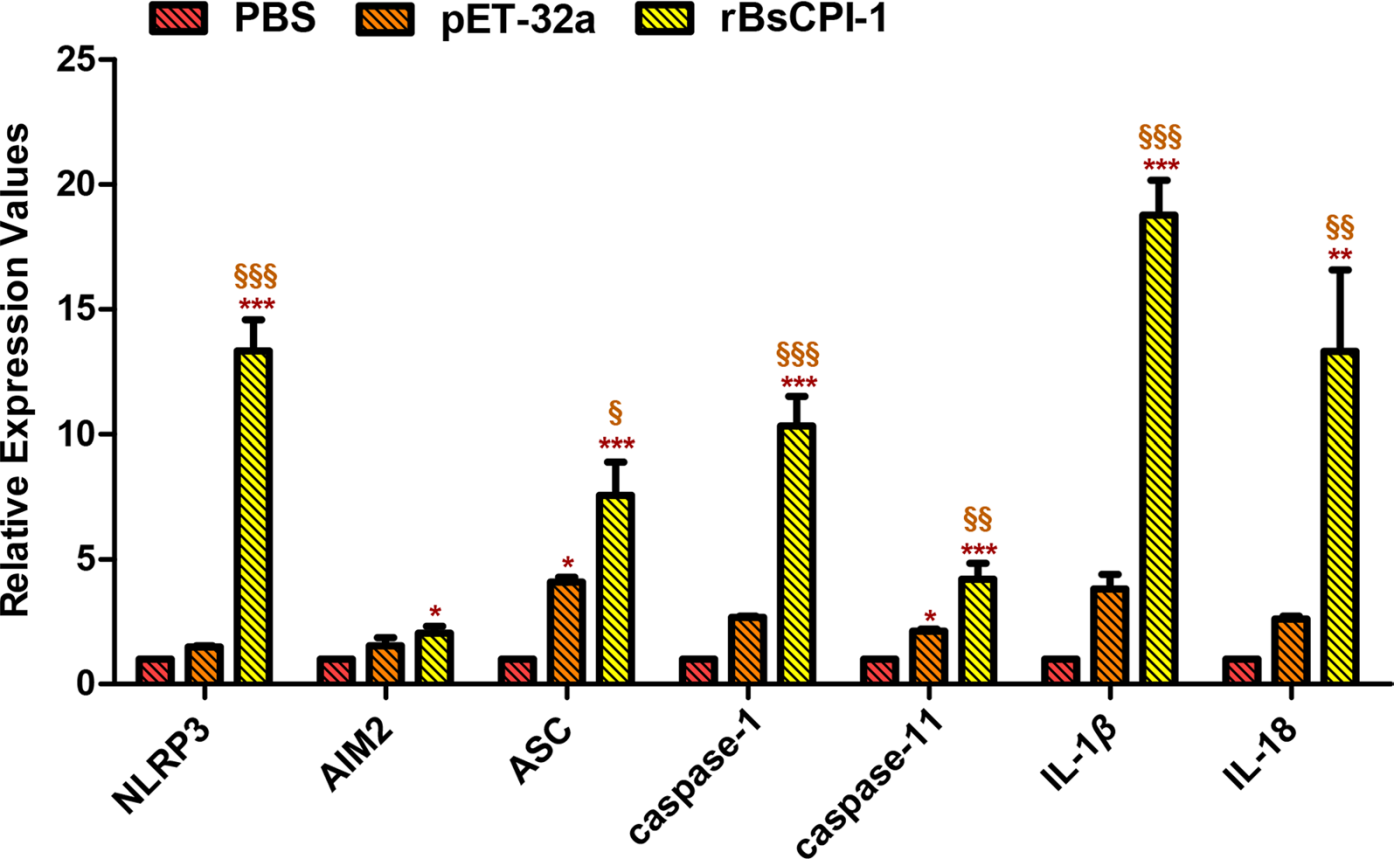
Analysis of the activation of the NLRP3 inflammasome in vivo. Data are shown as the mean ± SD of 3 replicates per group. Significant differences versus the control group are indicated by *P < 0.05, **P < 0.01, ***P < 0.001; significant differences versus the pET-32a group are indicated by ^§^*P* < 0.05, ^§§^*P* < 0.01, ^§§§^*P* < 0.001

We immunized mice by intraperitoneal injection with rBsCPI-1 for the in vivo experiments and extracted MDMs at 7 days after the last immunization. The qPCR results showed that rBsCPI-1 immunization induced the transcription levels of the NLRP3, AIM2, ASC, caspase-1, caspase-11, IL-1β and IL-18 compared to those in the control and pET-32a group (Fig. 5). Therefore, we preliminarily drew the conclusion that rBsCPI-1 induces NLRP3 inflammasome activation in MDMs.

## Discussion

Inflammation is a protective process that limits microbial infection. Innate immune cells recognize PAMPs or DAMPs through pattern recognition receptors, including membrane-bound TLRs and cytoplasmic NLRs, to regulate the occurrence and development of inflammatory responses.

Studies have shown that NLRs play a key role in inducing inflammatory responses to invading parasites during infection [20]. Different members of the NLR family respond to different stimuli; for example, ligand recognition by NLR family members such as NLRP1, NLRP3, NLRP6 and NLRC4 leads to inflammasome activation. Inflammasomes are an important part of the innate immune system and can detect microbial pathogens and activate inflammatory cytokines [21, 22]. *Neospora caninum* infection induces NLRP3 inflammasome activation [23]. NLRP1, NLRP3 and AIM2 inflammasomes are involved in the immune response during *Toxoplasma gondii* infection [24]. The expression of NLPR3 was found to be higher in the peritoneal macrophages of *Trichinella spiralis*-infected mice than in uninfected mice [25], a finding that was consistent with previous studies [26, 27]. The NLRP3 inflammasome is activated in response to *Leishmania* infection, and is an important host response for limiting parasite replication in vitro and in vivo [28]. A recent study showed that AIM2 can amplify signals of infection and trigger atypical NLRP3 activation [29], and another study showed that the inflammasome adaptor ASC is required for their assembly [30]. Once the inflammasome complex is assembled, pro-caspase-1/-11 can be cleaved into active caspase-1/-11, which further converts the pro-inflammatory cytokines IL-1β and IL-18 into mature active forms. Caspase-1 cleavage of GSDMD triggers cell pyroptosis [31–34]. Pyroptosis is characterized by continuous swelling of the cells until the rupture of the cell membrane, resulting in the release of cell contents and the activation of inflammatory reactions [34–37].

It is well known that parasite excretory–secretory (ES) proteins function as bioactive molecules that directly interact with the host immune system. In previous study, we successfully predicted and annotated the secretory proteome by using the whole-genome and transcriptome data of *B. schroederi* [38]. At the same time, we found that many of the previously reported proteases and protease inhibitors involved in the parasite–host interaction were also abundant in the secretory proteome of *B. schroederi*. We then cloned and expressed the CPI that is highly expressed in migratory larvae of *B. schroederi* (rBsCPI-1) and proved that rBsCPI-1 is a component of the ES proteins. We also found that rBsCPI-1 antigen was also recognized by serum from mouse infected with *B. schroederi* (unpublished findings). Therefore, in the present study we focused on analyzing whether ES protein and rBsCPI-1 have regulatory effects on the activation of inflammasomes.

 After co-incubating PBS (negative control), pET-32a, rBsCPI-1, BsES and Nigericin (positive control) with MDMs, we were able to detect activation of inflammasomes. The qPCR, western blotting and ELISA results showed that both rBsCPI-1 and BsES can significantly induce the high expression of NLRP3, caspase-1/-11 and ASC and further promote cleavage of the N-terminal of GSDMD while, at the same time, promoting the production and secretion of IL-1β and IL-18, thereby regulating the inflammatory response. We then analyzed the signaling pathway by which rBsCPI-1 regulates the NLRP3 inflammasome activation. Studies have shown that NLRP3 activation requires immune cells to be exposed to priming stimuli, such as TLRs or cytokine receptors. After initiation, NLRP3 is activated by certain stimuli, such as ion flux, ROS and lysosomal damage [39].

 ROS is involved in the regulation of many important cellular events, including proliferation, differentiation, immune response, cell growth and cell survival [40, 41]. ROS production has been identified as a key factor in NLRP3 inflammasome activation in parasite infections such as those by *Plasmodium falciparum* [42], *Trypanosoma cruzi* [43] and *Toxoplasma gondii* [44]. However, ROS-induced activation of NLRP3 inflammasome remains controversial [45–48]. TLR4 plays a key role in mediating ROS production [49]. Our previous experiments have confirmed that rBsCPI-1 can play an immunoregulatory role by being recognized by TLR4 on the surface of MDMs. Therefore, we speculated that rBsCPI-1 activates the NLRP3 inflammasome by binding to TLR4 and then inducing the high expression of intracellular ROS. To verify this speculation, we pre-blocked MDMs with TLR4 blocking antibody for 24 h, then incubated rBsCPI-1 with MDMs for 12 h; changes in ROS expression were detected in MDMs. The experimental results showed that the ability of rBsCPI-1 to induce ROS production decreased significantly when the TLR4 was blocked. On the other hand, we pre-incubated MDMs with a ROS inhibitor (NAC) for 4 h, and then co-incubated MDMs with rBsCPI-1. Western blotting results showed that the expression level of NLRP3 activated by rBsCPI-1 was significantly reduced with ROS inhibition, and even significantly lower than that in the control and NAC + PBS groups. We speculate that the reason for this may be that rBsCPI-1, as a CPI, negatively impacted the inflammatory caspases when ROS was inhibited, thereby further inhibiting the activation of the inflammasome. This led us to make a preliminary suggestion that rBsCPI-1 induces MDMs to generate high levels of ROS after binding to TLR4 on the surface of MDMs, and that ROS acts as an activator of NLRP3, thereby inducing the activation of NLRP3. In brief, rBsCPI-1 induces inflammasome assembly via the TLR4–ROS–NLRP3 pathway.

Studies have shown that the NLRP3 receptor as well as ASC and caspase-1/-11 are critical for the secretion of active factors that ultimately induce the production of ROS [50]. Therefore, ROS not only plays a key role in the activation of the inflammasome, but it is also a product of its own activation [51]. We pre-incubated MDMs with NLRP3 inhibitor (MCC950) for 4 h and then co-incubated them with rBsCPI-1. Changes in the expression level of ROS were detected. The level of ROS production in the MCC950 + CPI-1 group was significantly lower than that in the PBS + CPI-1 group. Therefore, our experimental results proved that ROS is both an NLRP3 activator and, to a certain extent, an NLRP3 activation product. We also detected the activation of caspase-1, IL-1β, IL-18 and pyroptosis marker cleaved* N*-terminal GSDMS induced by rBsCPI-1 after MDMs were pre-incubated with MCC950 Sodium. The experimental results showed that the activation levels of caspase-1, IL-1β, IL-18 and cleaved* N*-terminal GSDMS in the MCC950 + CPI-1 group were significantly lower than those in the PBS + CPI-1 group, but significantly higher than those in the control group. Therefore, our results preliminarily confirm that rBsCPI-1 induces pyroptosis by activating the NLRP3 inflammasome. We also performed recombinant protein immunization mice experiments, and the experimental results showed that rBsCPI-1 significantly induced the activation of NLRP3, caspase-1/-11, IL-1β and IL-18, thereby promoting the assembly of the NLRP3 inflammasome, which in turn is involved in the regulation of inflammatory response; these results are consistent with those of the in vitro experiments.

## Conclusions

We analyzed the effect of rBsCPI-1 in a mice model through in vitro and in vivo experiments and arrived at the following conclusions. rBsCPI-1 induces the activation of inflammasomes through the TLR4–ROS–NLRP3 signaling pathway. On the other hand, activation of the NLRP3 inflammasome further induces ROS expression. Inflammasomes induce cell pyroptosis and promote the production and secretion of the pro-inflammatory cytokines IL-1β and IL-18, thereby regulating the occurrence and development of inflammatory responses.

## Data Availability

The datasets used or analysed during the current study are available from the corresponding author on reasonable request.

## Abbreviations

*B. schroederi*: *Baylisascaris schroederi*
CPI: Cysteine protease inhibitors
NLRP3: Nucleotide-binding oligomerization domain, leucine-rich repeat and pyrin domain-containing 3
MDMs: Monocyte derived macrophages
ES: Excretory-secretory antigen
TLR4: Toll-like receptor 4
ROS: Reactive oxygen species
IL-1β: Interleukin-1 beta
IL-18: Interleukin-18
GSDMD: Gasdermin D
PRRs: Pattern recognition receptors
PAMPs: Pathogen-associated molecular patterns
DAMPs: Damage-associated molecular patterns
ASC: Apoptosis-associated speck-like protein containing CARD
AIM2: Absent in melanoma 2
LDH: Lactate dehydrogenase
NAC: N-Acetylcysteine
DCFH-DA: 2,7-Dichlorodi-hydrofluorescein diacetate
LPS: Lipopolysaccharide
NOD: Cytoplasmic nucleotide oligomerization domain
NLR: NOD-like receptor
NLRC4: Nucleotide-binding oligomerization domain, leucine-rich repeat and caspase recruitment domain-containing 4
